# Updated severity and prognosis score of pulmonary alveolar proteinosis: A multi-center cohort study in China

**DOI:** 10.3389/fmed.2023.1058001

**Published:** 2023-02-07

**Authors:** Jiu-Wu Bai, Jian-nan Huang, Shen-yun Shi, Ai Ge, Hai-wen Lu, Xiao-li Sun, Shu-yi Gu, Shuo Liang, Ke-bin Cheng, Xin-lun Tian, Yong-long Xiao, Kai-feng Xu, Jin-Fu Xu

**Affiliations:** ^1^Department of Respiratory and Critical Care Medicine, Shanghai Pulmonary Hospital, School of Medicine, Tongji University, Shanghai, China; ^2^Department of Respiratory and Critical Care Medicine, Peking Union Medical College Hospital, Chinese Academy of Medical Sciences & Peking Union Medical College, Beijing, China; ^3^Department of Respiratory and Critical Care Medicine, The Affiliated Drum Tower Hospital of Nanjing University Medical School, Jiangsu, China

**Keywords:** autoimmune pulmonary alveolar proteinosis, disease severity score, granulocyte-macrophage colony-stimulating factor, severity and prognosis score, whole lung lavage

## Abstract

**Background:**

The high-resolution computed tomography (HRCT) score is an important component of the severity and prognosis score of pulmonary alveolar proteinosis (SPSP). However, the HRCT score in SPSP only considers the extent of opacity, which is insufficient.

**Methods:**

We retrospectively evaluated HRCT scores for 231 patients with autoimmune pulmonary alveolar proteinosis (APAP) from three centers of the China Alliance for Rare Diseases. The SPSPII was created based on the overall density and extent, incorporating the SPSP. The severity of APAP patients was assessed using disease severity scores (DSS), SPSP, and SPSPII to determine the strengths and weaknesses of the different assessment methods. We then prospectively applied the SPSPII to patients before treatment, and the curative effect was assessed after 3 months.

**Results:**

The HRCT overall density and extent scores in our retrospective analysis were higher than the extent scores in all patients and every original extent score severity group, as well as higher related to arterial partial oxygen pressure (PaO_2_) than extent scores. The mild patients accounted for 61.9% based on DSS 1–2, 20.3% based on SPSP 1–3, and 20.8% based on SPSPII 1–3. Based on SPSP or SPSPII, the number of severe patients deteriorating was higher in the mild and moderate groups. When applied prospectively, arterial PaO_2_ differed between any two SPSPII severity groups. The alveolar-arterial gradient in PaO_2_ (P[A-a]O_2_), % predicted carbon monoxide diffusing capacity of the lung (DLCO), and HRCT score were higher in the severe group than in the mild and moderate groups. After diagnosis, mild patients received symptomatic treatment, moderate patients received pure whole lung lavage (WLL) or granulocyte-macrophage colony-stimulating factor (GM-CSF) therapy, and severe patients received WLL and GM-CSF therapy. Importantly, the SPSPII in mild and severe groups were lower than baseline after 3 months.

**Conclusion:**

The HRCT density and extent scores of patients with APAP were better than the extent score. The SPSPII score system based on smoking status, symptoms, PaO_2_, predicted DLCO, and overall HRCT score was better than DSS and SPSP for assessing the severity and efficacy and predicting the prognosis.

**Trial registration:**

ClinicalTrial.gov, identifier: NCT04516577.

## Introduction

Pulmonary alveolar proteinosis (PAP) is a rare lung syndrome characterized by the intra-alveolar accumulation of surfactant lipids and proteins. PAP is primarily divided into three groups: congenital PAP, secondary PAP, and autoimmune PAP (APAP) (1). The prevalence of APAP is 0.1 per 100,000 of the population and accounts for ~90% of all PAP cases (1, 2). Granulocyte-macrophage colony-stimulating factor (GM-CSF) antibody levels significantly increase in the serum and bronchoalveolar lavage fluid (BALF) of APAP patients (3) and have a high affinity for GM-CSF, decreasing its activity (4).

In a previous study, 7% of PAP patients went into spontaneous remission, indicating that not all patients require whole lung lavage (WLL) (5). In 2008, a disease severity score (DSS) was first proposed to assess PAP severity, divided into five grades based on symptoms and arterial partial pressure of oxygen (PaO_2_) (6). In 2016, our team proposed the severity and prognosis score for PAP (SPSP) based on smoking status, symptoms, PaO_2_, high-resolution computed tomography (HRCT) score, and the carbon monoxide diffusing capacity of the lung (DLCO) (7). In the SPSP, the HRCT score is the area based on the percentage extent of lung opacity in the corresponding lung region, not uninvolving the density of lesions. In 2017, Tokura et al. (8) suggested that increased parenchymal opacity be evaluated based on its density and extent. Therefore, updating the SPSP based on the density and extent of opacity with imaging systems is appropriate.

## Methods

### Study population

APAP patients aged between 18 and 80 years were enrolled at three hospitals in China: Shanghai Pulmonary Hospital, Peking Union Medical College Hospital, and Nanjing Drum Tower Hospital of China Alliance for Rare Diseases. This study was divided into two parts. In the first part, the data from 231 patients were collected and retrospectively cross-sectionally analyzed between January 2010 and December 2019. All patients completed at least a 6-month follow-up. The prognosis was assessed as improving, stable, or deteriorating by the physician based on the reviewed chest HRCT, many of which were not reviewed at the original hospital, and specific follow-up HRCT scores after treatment were not accessed. In the second part, 36 newly diagnosed patients were enrolled in a prospective study according to the newest severity assessment criteria between January 2020 to October 2021. They were provided with appropriate treatment and a 3 month follow-up. A total of 31 patients completed treatment and followed-up, and five patients withdrew during follow-up. This study was approved by the Ethics Committees of Peking Union Medical College Hospital (JS-2639), Shanghai Pulmonary Hospital (K19-133), and Nanjing Drum Tower Hospital (2019–106-1) and registered on the Clinical Trials database (ID: NCT04516577).

The eligibility criteria were selected according to Ben-Dov et al. (9): (1) histopathologic findings of specimens obtained by open or transbronchial lung biopsy confirmed by testing for amorphous periodic acid-schiff positive granules, (2) a typical milk-like BALF appearance with lamellar bodies visible on electron microscopy, (3) ground-glass opacity or a crazy-paving pattern on HRCT, and (4) a positive serum GM-CSF antibody test indicating an elevated serum GM-CSF antibody level. The retrospective patients were diagnosed with APAP by clinical synthetic judgment based on featured HRCT with typical pathology and BALF evidence (*n* = 86), with pathology evidence (*n* = 38), or with BALF evidence (*n* = 107). In the prospective study, APAP diagnosis was based on typical pathology and BALF.

### Interview questionnaire

A standardized protocol was used to obtain informed consent from each participant during a medical visit. The interview questionnaire used included questions on the following topics: anthropometric information (i.e., age and sex), smoking status (e.g., smoker or never smoked), and clinical manifestation (e.g., the onset of symptoms, time of symptom onset, and symptoms).

Forced vital capacity (FVC), forced expiratory volume in 1 s (FEV_1_), and DLCO data were presented as percentages of predicted values (% predicted). Arterial blood measurements were performed on samples obtained while the patients were breathing room air at rest in the supine position, including PaO_2_, and alveolar–arterial gradient in PaO_2_ (P[A-a] O_2_).

### Severity assessment of patients

Based on the recommendations of Inoue et al. (6), DSS categories included: 1 = asymptomatic and PaO_2_ ≥ 70 mm Hg; 2 = symptomatic and PaO_2_ ≥ 70 mm Hg; 3 = 60 ≤ PaO_2_ < 70 mm Hg; 4 = 50 ≤ PaO_2_ < 60 mm Hg; 5 = PaO_2_ < 50 mm Hg. The patients were divided into mild (DSS 1–2), moderate (DSS 3), and severe (DSS 4–5) groups according to Leth et al. (9).

Chest HRCT were examined and interpreted independently by two chest physicians using imaging systems. The mean values obtained from the two readers were used for analysis. We selected the HRCT grades in four representative regions: the aortic arch, the tracheal carina, the convergence of the left and right inferior lung veins, and above the diaphragm. The extent score of lung opacity was estimated using a five-point scale: 0 = no opacity; 1 = opacity involving ≤25% of a region of hemithorax; 2 = 26–50%; 3 = 51–75%; 4 = >75%. The average density score of lung opacity in four representative regions was estimated using a three-point scale: 1 = density of opacity ≤ − 400; 2 = > −400 and ≤ −100; 3 = > −100. The overall score was updated based on the density score multiplied by the extent score and included a six-point scale: 0 = no opacity; 1 = ≤10; 2 = 11–20; 3 = 21–30; 4 = 31–40; 5 = >40.

The original SPSP included smoking statues (0 = never smoker; 1 = smoker); symptoms (0 = No; 1 = Yes); PaO_2_ (0 = ≥80 mm Hg; 1 = ≥60 mmHg and < 80 mm Hg; 2 = <60 mm Hg); HRCT extent score of lung opacity (1 = ≤8; 2 = >8 and ≤ 16; 3= >16 and ≤ 24; 4= >24); and % predicted DLCO (0 = ≥80%; 1 = ≥60 and < 80%; 2 = <0%) (7). SPSP was updated to SPSPII based on the overall HRCT chest score. Patients were divided into mild (SPSP or SPSPII 1–3), moderate (SPSP or SPSPII 4–6) and severe (SPSP or SPSPII ≥7).

### Statistics

In the retrospective study, patient HRCT was evaluated based on the extent score and overall score with the self-contrasted method. The severity was assessed by three methods (DSS, SPSP, and SPSPII) and divided into three levels (mild, moderate, and severe). Patient prognoses were classified as improved, stable, or deteriorated based on their follow-up results. In the prospective study, patients were assessed by the SPSPII and divided into three severity levels. The improvement group (SPSPII was decreased) and non-improvement group (SPSPII was not decreased) were according to the change of SPSPII between the baseline and after 3 months. The statistical software SPSS (v.19.0; IBM, Armonk, NY, USA) and GraphPad Prism (v.5; GraphPad Software, San Diego, CA, USA) were used for all statistical analyzes and graph plotting, respectively. The data were tabulated as means and standard deviations (SDs) for quantitative variables or as absolute numbers and percentages for qualitative variables. The Kolmogorov–Smirnov test was used to assess the distribution for each variable. In bivariate analyzes, the Student’s t-test for independent variables was used for normally distributed variables, and the Mann–Whitney U test was used for non-normally distributed variables. Qualitative variables were compared using the Chi-square test. All results with *p* < 0.05 were considered statistically significant.

## Results

**Figure 1:**
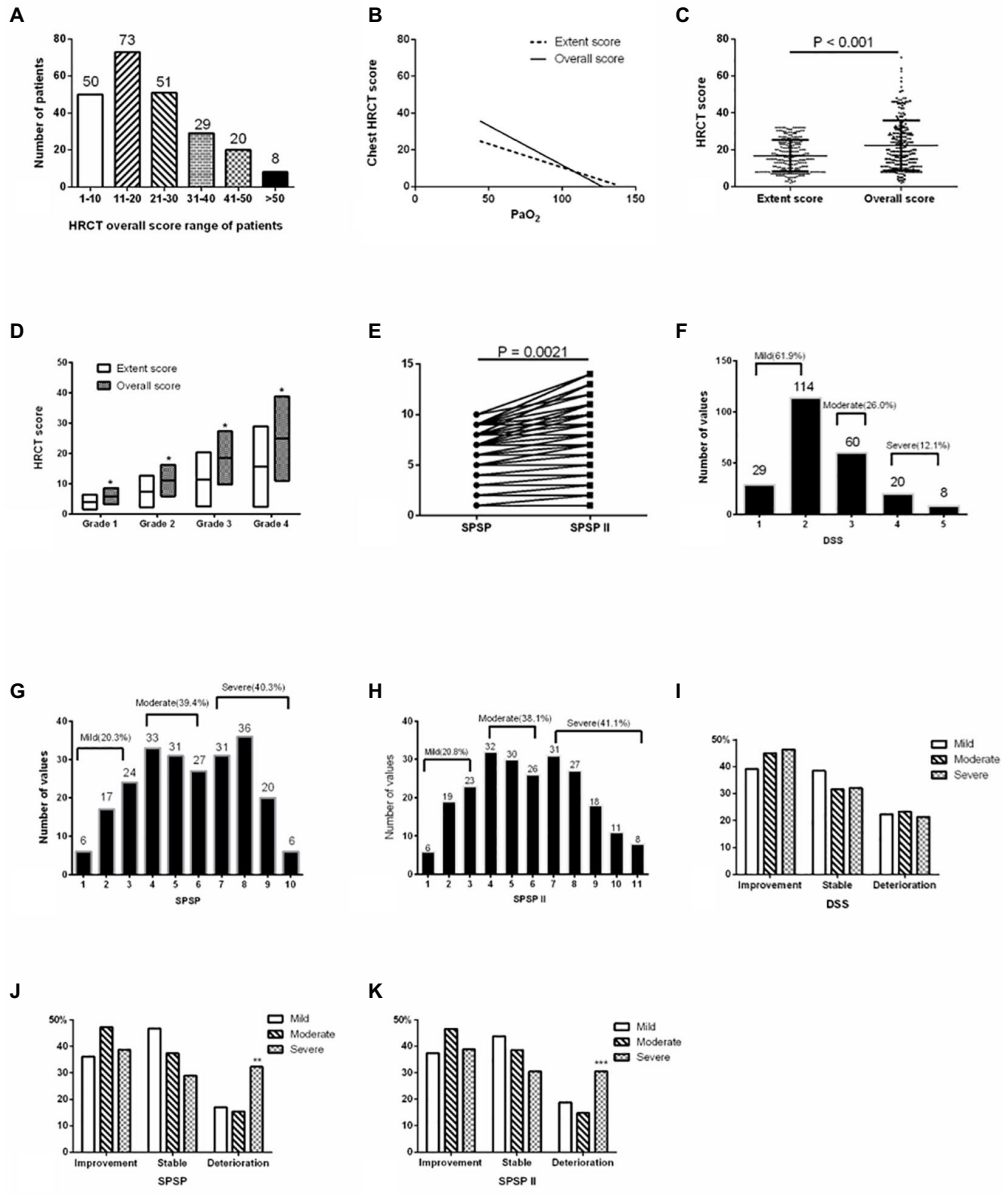
Severity assessment of APAP patients in the retrospective study. **(A)** Overall HRCT score of patients. **(B)** The correlation between HRCT score and PaO_2_ (overall score of HRCT and PaO_2_ [*r* = −0.4537, *p* < 0.001]; extent score of HRCT and PaO_2_ [*r* = −0.4366, *p* < 0.001]). **(C)** Comparison of the extent and overall scores. **(D)** Comparison of scores in every grade between extent and overall score (grade 1: ≤8; grade 2: >8 and ≤ 16; grade 3: >16 and ≤ 24; grade 4: >24). **(E)** SPSP and SPSPII of patients. **(F)** DSS-based severity classification. **(G)** SPSP-based severity classification. **(H)** SPSPII-based severity classification. **(I)** Patient prognosis with DSS-based severity. **(J)** Patient prognosis with SPSP-based severity. **(K)** Patient prognosis with SPSPII-based severity. APAP, Autoimmune pulmonary alveolar proteinosis; DSS, Disease severity score; HRCT, High-resolution computed tomography; *p < 0.001; ** the proportion of deterioration in the severe group was higher than in the mild–moderate groups (*p* = 0.004); ***the proportion of deterioration in the severe group was higher than in the mild–moderate groups (*p* = 0.013).

The overall HRCT score of patients in the retrospective study was shown in Figure 1A. This presented a negative relation to PaO_2_ (*r* = −0.4537, *p* < 0.001), and the coefficient was higher than that between the extent score of HRCT and PaO_2_ (*r* = −0.4366, *p* < 0.001; Figure 1B). The overall score of HRCT was higher than the extent score in all patients and every original extent score severity group (all *p* < 0.001; Figures 1C,D; Table 1). Updated SPSP (SPSPII) scores based on the overall score were shown in Table 2; these were higher than the SPSP scores (Figure 1E; *p* = 0.0021).

**Table 1:** Different HRCT score of patients with autoimmune APAP.

HRCT grade	1	2	3	4
Primal HRCT score scope	≤8	>8 & ≤16	>16 & ≤24	>24
*N* (%)	54 (23.4)	66 (28.6)	59 (25.5)	52 (22.5)
Extent score	6.4 ± 1.6	12.6 ± 2.3	20.4 ± 2.5	28.9 ± 2.4
Overall score	8.6 ± 3.2	16.3 ± 5.9	27.3 ± 9.8	38.8 ± 11.0
Number of score increases (%)	24(44.4)	25(37.9)	35(59.3)	29(55.8)
*p* valve	**<0.001**	**<0.001**	**<0.001**	**<0.001**

APAP, Autoimmune pulmonary alveolar proteinosis; HRCT, High resolution computed tomography.

**Table 2:** Scoring Standards in SPSPII.

Score	Smoking	Symptom	PaO_2_, mmHg	DLCO, %predicted	HRCT overall score
0	No	No	≥80	≥80	/
1	Yes	Yes	≥ 60 & < 80	≥ 60 & < 80	≤10
2	/	/	< 60	< 60	> 10 & ≤ 20
3	/	/	/	/	>20 & ≤ 30
4	/	/	/	/	> 30 & ≤ 40
5	/	/	/	/	> 40

DLCO, Carbon monoxide diffusing capacity; PaO2, Arterial partial pressure of oxygen; PAP, Pulmonary alveolar proteinosis; SPSP, Severity and prognosis score of PAP.

Based on DSS, the proportions of the different severities were 61.9% (DSS1, 29, 12.6%; DSS2, 114, 49.4%; mild), 26.0% (DSS3, moderate), and 12.1% (DSS4-5, severe; Figure 1F). Based on SPSP and SPSPII, the proportions of the different severity groups were shown in Figures 1G,H. The specific treatment and follow-up outcomes of patients were shown in Table 3. Based on DSS, the patient prognosis did not differ among severity groups (Figure 1I). However, based on SPSP or SPSPII, the proportion of patients deteriorating in the severe group was higher than in the mild and moderate groups (Figures 1J,K).

**Table 3:** Treatment and prognosis of patients in retrospective section.

	*N* (%)	Treatment				Prognosis		
		WLL	Nebulization of GM-CSF	Subcutaneous of GM-CSF	Symptomatic and other treatment	Improvement	Stable	Deterioration
DSS								
Mild	143 (61.9)	21	24 (WLL: 8)^a^	28 (WLL: 2)	69	56	56	31
Moderate	60 (26.0)	11	12 (WLL: 2)	13 (WLL: 1)	26	27	19	14
Severe	28 (12.1)	9	2 (WLL: 1)	6 (WLL: 2)	8	13	9	6
SPSP								
Mild	47 (20.3)	4	5 (WLL: 1)	2	36	17	22	8
Moderate	91 (39.4)	8	13 (WLL: 2)	25 (WLL: 2)	45	43	35	13
Severe	93 (40.3)	29	20 (WLL: 8)	30 (WLL: 3)	10	36	27	30
SPSPII								
Mild	48 (20.8)	4	4 (WLL: 1)	4	36	18	21	9
Moderate	88 (38.1)	11	13 (WLL: 3)	27(WLL: 2)	39	41	34	13
Severe	95 (41.1)	26	21 (WLL: 7)	16 (WLL: 3)	38	37	29	29

DSS, Disease severity score; GM-CSF, Granulocyte-macrophage colony-stimulating factor; WLL, Whole lung lavage.

a The patients had the history of whole lung lavage.

**Figure 2:**
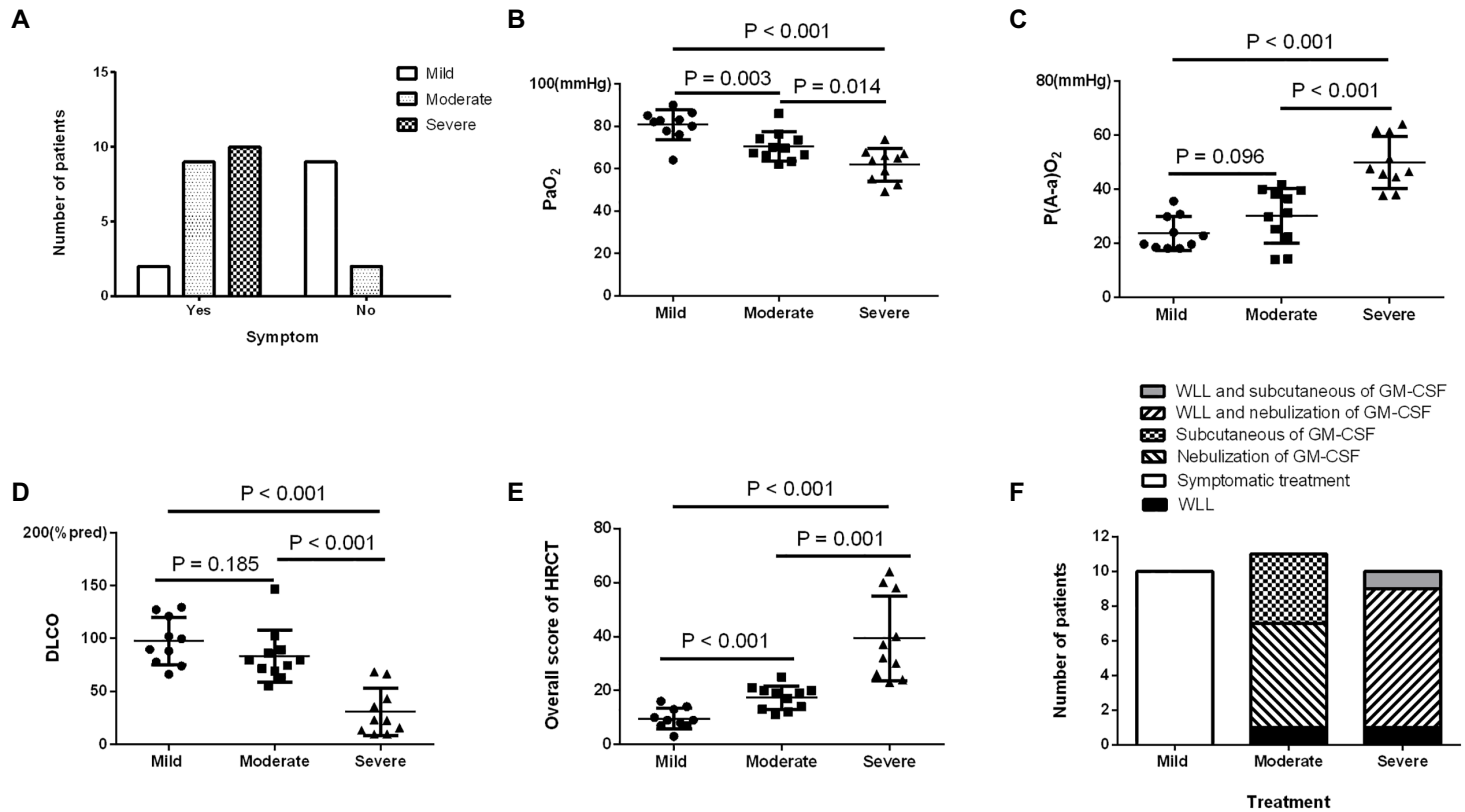
The characteristics and prognosis of patients with different SPSPII-based severities in the prospective study. **(A)** Symptoms**. (B)** PaO_2_. **(C)** P(A-a) O_2_. **(D)** DLCO. **(E)** Overall HRCT score. **(F)** The treatment options of patients with different severities.

In the prospective study, the number of patients in the different SPSPII-based severity groups was 10 (mild), 11 (moderate), and 10 (severe). No apparent differences existed in age, sex, smoking history, onset age, or time of symptom onset among severity groups (all *p > 0.05*). Moderate and severe patients presented more symptoms than mild patients (*p* = 0.046; Figure 2A). However, PaO_2_, P[A-a] O_2_, % predicted DLCO, and overall HRCT score among different severity groups were shown in Figures 2B–E.

**Figure 3:**
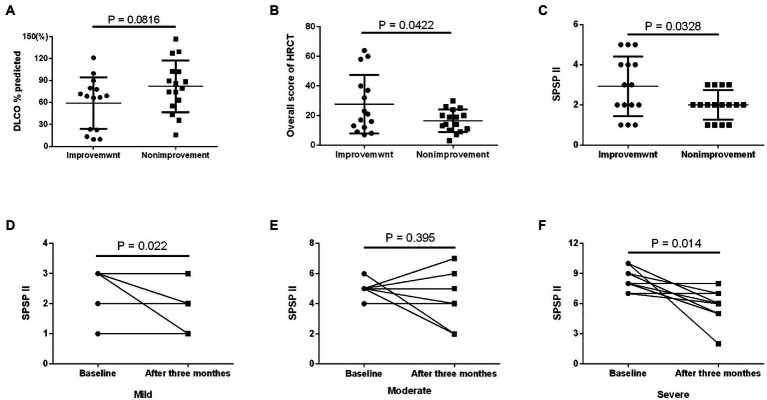
Comparison of patients at baseline and after 3 months in the prospective study. Changes in some variables of 31 patients at baseline and after 3 months: **(A)** % predicted DLCO; **(B)** overall score of HRCT; **(C)** SPSPII. Changes in SPSPII of patients at baseline and after 3 months in different severity; **(D)** mild group; **(E)** moderate group; **(F)** severe group.

Based on the SPSPII, the patients in different severities received different treatment: symptomatic treatment in mild patients, WLL or GM-CSF therapy based on their willingness and consent in moderate patients, WLL and GM-CSF therapy in severe patients (Figure 2F). The SPSPII of all patients after 3 months (4.0 ± 2.2) was lower than at baseline (5.2 ± 2.5; *p* = 0.002). According to the change of SPSPII between baseline and after 3 months, these patients were divided into two groups: improvement group (SPSPII decreasing, 15) and non-improvement group (stable, 2; SPSPII increasing, 14). The sex, age, smoking history, symptoms, onset of symptoms, time of symptom onset, PaO_2_ and P[A-a] O_2_ were similar between improvement group and non-improvement group (all *p > 0.1*). The % predicted DLCO, overall score of HRCT and SPSPII between baseline and after 3 months were shown in Figures 3A–C. The change of SPSPII in different severities at baseline and after 3 months were shown in Figures 3D–F.

## Discussion

APAP is an autoimmune disease of the lungs, (3) and similar to sarcoidosis, some patients experience spontaneous remission, (10, 11) and do not require WLL therapy. Inoue et al. (6) proposed the DSS to assess PAP severity in patients based on two criteria: symptoms and PaO_2_. DSS was simple, but relatively limited in assessing the efficacy and prognosis in patients (7). In 2016, we proposed SPSP to evaluate the severity and predict patient prognosis based on smoking status, symptoms, PaO_2_, HRCT extent score, and % predicted DLCO to some extent (7). In SPSP, the HRCT score was the percentage extent of lung opacity in the corresponding lung region, not uninvolving the density of lesions. However, while the extent of opacity was not significantly improved, density decreased in the HRCT of some PAP patients during follow-up during actual medical procedures. In this study, the overall score was based on the density score multiplied by the extent score, and presented higher correlation to PaO_2_ than simple extent score. Based on the overall HRCT score, SPSPII may be superior to the SPSP to evaluate the efficacy of patients before and after treatment. In addition, SPSPII was more beneficial to predict the prognosis of patients than DSS, as well as a better way to assess patient severity and prognosis.

Symptoms were subjective based on how the patient feels and may reflect severity to some extent. Previous studies have shown that some APAP patients were asymptomatic, (6, 12, 13) which may reflect the tolerance of patients with mild disease. PaO_2_ was a more objective indicator for assessing the severity of diffuse lung disease. In a previous study, the PaO_2_ of smokers was lower than nonsmokers in the general population (14). PAP patients with a smoking history had lower PaO_2_ than patients who had never smoked, (7) and the proportion of current smokers was significantly higher in the high DSS (DSS3-5) group than in the low DSS (DSS1-2) group (15). For PAP patients, the most common abnormality in pulmonary function tests was a decrease in % predicted DLCO, (10) which had a stronger correlation with PaO_2_ than FVC and FEV_1_ (7).

The degree of lesion abnormality on HRCT scans can be evaluated by visual assessment or objective quantification (16, 17). Ground-glass opacities and symmetric diffuse bilateral lung involvement were the predominant HRCT presentation of PAP (18, 19). In our previous study, the extent score of lung opacity was an SPSP component and negatively correlated with PaO_2_ (7). In 2017, Tokura et al. (8) suggested that increased parenchymal opacity be evaluated based on its density and extent. The visual score has been proposed to assess the severity of PAP patients based on evaluating the intensity and extent of opacities in HRCT (8, 20). However, this visual score was subjective and not completely accurate. Therefore, in this study, imaging systems were used to evaluate the intensity and extent of the opacities independently by two chest physicians, providing greater accuracy than the visual score.

The DSS 1–2 patients should be advised to regularly reassessment of symptoms, arterial blood gas analyzes, lung function tests, and chest X-rays by Leth et al. (9). A commonality (PaO_2_ ≥ 70 mm Hg) existed between DSS 1 and DSS 2. DSS 2 patients were considered mildly to moderately affected by Leth et al. (9). In this study, the DSS 2 patients accounted for 49.4%. What percentage of patients would further worsen and require treatment in the future was not clear. If mild patients include DSS1-2 and accounted for 61.9%, most patients would experience disease progression and require further treatment. Therefore, DSS was relatively simple and limited in clinical assessment of patient severity and choosing appropriate therapeutic schedule. When using SPSP or SPSPII, mild patients accounted for a little over 20%, indicating that nearly 80% of PAP patients will require treatment, this situation was more consistent with the actual treatment of patients with PAP. Deterioration was more likely to occur in severe patients than in mild and moderate patients classified based on SPSP or SPSPII. Therefore, SPSP and SPSPII appear superior to DSS for assessing the PAP severity and predicting patient prognosis.

Between improvement and non-improvement group at baseline and 3 months later, the patients in improvement group presented higher overall score of HRCT and SPSPII. This result may be because most patients with a high SPSPII received appropriate treatment, resulting in illness improvement. In the prospective study, mild patients were not given WLL or GM-CSF therapy, but their prognosis did not deteriorate. Ten moderate patients received GM-CSF therapy, and the SPSPII of more than 70% of patients was not decreased. This result may be related to finite-duration treatment with GM-CSF therapy. In previous studies, after 6 months of treatment, regardless of the treatment (subcutaneous injection or nebulized inhalation), GM-CSF showed significant improvement in % predicted DLCO and P[A-a] O_2_ (21, 22). Severe patients received WLL and GM-CSF therapy, and their condition was improved or stable.

This study was primarily limited in two aspects: there was no uniform standard on the treatment of patients in the retrospective study, and the number of cases was limited and the follow-up time was shorter in the prospective study. The retrospective study results indicated that SPSPII was the superior predictor, and the prospective study results largely accorded with this. Nevertheless, further studies of the SPSPII with larger sample sizes are required to confirm our findings.

## Conclusion

The density and extent HRCT score of APAP patients was superior to the extent score. SPSPII, based on smoking status, symptoms, PaO_2_, % predicted DLCO, and the overall HRCT score, was better at assessing the severity and efficacy and predicting prognosis than DSS and SPSP.

## Data availability statement

The original contributions presented in the study are included in the article/Supplementary material, further inquiries can be directed to the corresponding authors.

## Ethics statement

This study was, respectively, approved by the Ethics Committee of Peking Union Medical College Hospital, Shanghai Pulmonary Hospital and Nanjing Drum Tower Hospital. The patients/participants provided their written informed consent to participate in this study.

## Author contributions

J-WB, K-FX and J-FX made substantial contributions to the concept and design of the work. J-WB, J-NH and SYS conducted statistical analysis. AG, H-WL, X-LS, S-YG, SL, K-BC and X-LT made substantial contributions to acquisition of data. J-WB and J-FX drafted the initial manuscript. K-FX and Y-LX reviewed and revised the manuscript. All authors read and approved the final manuscript.

## Funding

This work was supported in part by grants from the Advanced and Appropriate Technology Promotion Project of Shanghai Municipal Health Commission (2019SY055) and Prevention and Treatment of Common and Frequent Diseases of National Key Research and Development Program Special project (2021YFC2500700).

## Conflict of interest

The authors declare that the research was conducted in the absence of any commercial or financial relationships that could be construed as a potential conflict of interest.

## Publisher’s note

All claims expressed in this article are solely those of the authors and do not necessarily represent those of their affiliated organizations, or those of the publisher, the editors and the reviewers. Any product that may be evaluated in this article, or claim that may be made by its manufacturer, is not guaranteed or endorsed by the publisher.
